# Supplementary material for: Gender disparities and barriers to access and use of essential health services in Ethiopia: Designing primary health care through gender lens
Source: PLOS Glob Public Health. 2025 Jun 20;5(6):e0004813. doi: 10.1371/journal.pgph.0004813 (PMC12180718; doi:10.1371/journal.pgph.0004813)
Supplement: Attachment — Submitted filename: Point by point response.pdf [file pgph.0004813.s001.pdf]

Point by point response

We've checked your submission and before we can proceed, we need you to address the following issues:

1. Please ensure that the Author List in your manuscript file matches the Author List in the online submission form, including initials and order. Currently, author "Yohannes Mehretie" name listed in manuscript file is different from online submission form.

- **Corrected as "Yohannes Mehretie"**

  Please use either initials or full names in both the manuscript and online submission form.

2. Please complete all items on the Human Participants Research Checklist that are relevant for your submission, by following this link: http://journals.plos.org/plosone/s/file?id=dc11/PLOSOne_Human_Subjects_Research_Checklist.docx. There may be overlap between the checklist items and other queries listed here; please address any duplicated queries both in your response email and on the checklist itself. Upload the completed Human Participants Research checklist as file type "Other" when you re-submit your manuscript. This document is for internal journal use only and will not be published if your article is accepted. The requested information will help us to assess whether your submission complies with PLOS's policies and adheres to applicable reporting standards. Note that your manuscript may be rejected if you provide incomplete or inadequate responses to the checklist questions and that changing the 'Section/Category' of your article does not affect this requirement.

- **Corrected: the checklist is completed. Thank you so much!**

Authors,
