# Supplementary material for: Gender disparities and barriers to access and use of essential health services in Ethiopia: Designing primary health care through gender lens
Source: PLOS Glob Public Health. 2025 Jun 20;5(6):e0004813. doi: 10.1371/journal.pgph.0004813 (PMC12180718; doi:10.1371/journal.pgph.0004813)
Supplement: Attachment — Submitted filename: Response to Reviewers.pdf [file pgph.0004813.s002.pdf]

# Point-by-point rresponse to Reviewers'

Gender disparities and barriers to access and use of essential health service in Ethiopia: designing primary health care through gender lens: PLOS Global Public Health (PGPH-D-24-02755)

| Section | Comment | Response to reviewers |
|---|---|---|
| General comment | Thank you for submitting your manuscript to PLOS Global Public Health. After careful consideration, we feel that it has merit but does not fully meet PLOS Global Public Health's publication criteria as it currently stands. Therefore, we invite you to submit a revised version of the manuscript that addresses the points raised during the review process. | *We are so grateful for the constructive comments and duly addressed all the comments from reviewers and editors for the betterment of our article.* |
| Theoretical framework | The paper brings a description of the current challenges. It includes some contextual factors and recognition of systemic and gendered barriers. There are elements that need addressing. Given the paper is about a gender lens there is no mention of the gender framework or theories used to inform the thinking. Whilst there is mention that gender is a construct there is no critical underpinning. The subject matter is gender but there is no gender theory. Given the gender theories in the literature I feel this is a missing aspect of the paper | *Thanks, we used multifaceted theoretical frameworks and qualitative (interpretive) philosophical underpins. We have breakdown sub-section "study theoretical underpinning and design". see track change in the method section; page 6-7* |
| Methods | The sample information is incomplete and I am not clear on the inclusion / exclusion criteria. | Thanks, we have addressed the concern in the methods section "sampling methods". See track change page 7. |
| Results | The results section could be more clear. Whilst there are themes noted, it is not clear how many contributors contributed to each themes. The quotes show the themes, which may be enough. However, there is nothing then constructed from themes. | *Thanks, we have presented the finding based on the USAID's transform primary healthcare gender analysis domains.* |
| Discussion | The discussion section could be more clear and build on the citations. The grammar is not clear and in particular lines 14-26 need to be rewritten to give more clarity. It does not make enough sense. | Thanks, necessary revisions made |
| Conclusion | Communities' culture' should be community's culture or communities' culture. | *thanks, addressed "communities' culture*" |
| Language | I suggest running this through a grammar check and addressing the conceptual gaps and clarity needed as noted | *Thanks, Addressed grammar issues.* |
| Information | rebuttal letter that responds to each point raised by the editor and reviewer(s). You should upload this letter as a separate file labeled 'Response to Reviewers'. | *Thanks, we have prepared the point-bi point response along with ''Revised Manuscript with Track Changes'' and "manuscript"* |
| | A marked-up copy of your manuscript that highlights changes made to the original version. You should upload this as a separate file labeled 'Revised Manuscript with Track Changes'. | *For visibility of the amendment. We have submmitted the revised version "'Revised Manuscript with Track Changes'" along with the cleaned manuscript and necessary documents* |
| | An unmarked version of your revised paper without tracked changes. You should upload this as a separate file labeled 'Manuscript' | *Revised version label as "Manuscript"* |

Thanks,

Authors,
