# Supplementary material for: Gender disparities and barriers to access and use of essential health services in Ethiopia: Designing primary health care through gender lens
Source: PLOS Glob Public Health. 2025 Jun 20;5(6):e0004813. doi: 10.1371/journal.pgph.0004813 (PMC12180718; doi:10.1371/journal.pgph.0004813)
Supplement: Attachment — Submitted filename: Response_to_reviewers_auresp_2.pdf [file pgph.0004813.s003.pdf]

Point-by-point response to reviewers

Gender disparities and barriers to access and use of essential health service in Ethiopia: designing primary health care through gender lens
PLOS Global Public Health: PGPH-D-24-02755R1

Content	Comments from reviewers	*Response from authors*
	Editorial team	
General comment	The reviewers have raised a few remaining of concerns. They feel that the discussion should contain a deeper reflection, given the sensitive nature of the topic. Could you please carefully revise the manuscript to address all comments raised?	*We are so grateful for the insightful and helpful review feedbacks from reviewers and editorial team. We have addressed the concerns as per suggestion and see track change.*
	Reviewer #1:	
General comment	I congratulate the authors for carrying out the study in a careful and comprehensive manner. The article is structured and written in clear language. It addresses an important topic to be discussed at a global level.	*Thank you so much for the unreserved comments and contribution for the betterment of our manuscript.*
Discussion	I only present recommendations regarding the structuring of the discussion, which had already been requested for corrections. I recommend that the reflections be deepened, given that the authors address a sensitive topic such as gender inequality in terms of access to health care. There is still a lack of clarity regarding what constitutes a result and what the scientific literature reports. It would be interesting for the authors to make comparisons, including mentioning the scenario in which they are making the comparisons.	*Many thanks for the comment and concern we have amended the discussion and added more references to enrich the discussion and improve its quality as well. All comments were so helpful for our work and grateful for that. See track change.*
Conclusion	I also recommend more practical actions as an attempt to change this scenario, not just generalizing with national policies.	*Thank you so much we have focused on the gender-integration health service in the PHC system of Ethiopia. See track change*

Regards,

Authors,
